# Temporal Dynamics of Clinical Response Following Fecal Microbiota Transplantation in Dogs with Chronic Enteropathy

**DOI:** 10.3390/ani16172786

**Published:** 2026-09-04

**Authors:** Maria Chiara Sabetti, Rachel Pilla, Francesca Fidanzio, Carla Giuditta Vecchiato, Giorgia Galiazzo, Alessandro Tirolo, Michela Ablondi, Silvia Scorza, Mariangela Colosini, Jan S. Suchodolski, Andrea Corsini

**Affiliations:** 1 Department of Veterinary Science, University of Parma, Strada del Taglio, 10, 43126 Parma, Italy; mariachiara.sabetti@unipr.it (M.C.S.); alessandro.tirolo@unipr.it (A.T.); michela.ablondi@unipr.it (M.A.); silvia.scorza@studenti.unipr.it (S.S.); mariangela.colosini@unipr.it (M.C.); andrea.corsini@unipr.it (A.C.); 2 Department of Veterinary Pathology, Hygiene and Public Health, University of Milan, Via dell’Università, 6, 26900 Lodi, Italy; rachel.pilla@unimi.it; 3 Gastrointestinal Laboratory, Texas A&M University, College Station, TX 778432, USA; jsuchodolski@cvm.tamu.edu; 4 Department of Veterinary Medical Sciences, Alma Mater Studiorum-University of Bologna, Via Tolara di Sopra 50, Ozzano dell’Emilia, 40127 Bologna, Italy; carla.vecchiato2@unibo.it; 5 Veterinary Clinical Practice, 45100 Rovigo, Italy; giorgiagali@gmail.com

**Keywords:** clinical diary, clinical score, CIBDAI, FMT, owner-reported monitoring, longitudinal follow-up, treatment response

## Abstract

Fecal microbiota transplantation (FMT) is a promising adjunctive treatment for dogs with chronic enteropathy (CE), but response is usually assessed at scheduled follow-up visits, potentially overlooking day-to-day fluctuations. This prospective observational study evaluated temporal response after a single FMT administered by retention enema using daily owner-completed Canine Inflammatory Bowel Disease Activity Index (CIBDAI) monitoring. Fourteen dogs with CE refractory or incompletely responsive to dietary management were enrolled. CIBDAI scores were assigned by a veterinarian at baseline and on days 7 and 30, while owners completed a daily clinical diary for 30 days. Changes over time were assessed using the Friedman test, longitudinal trends using a generalized additive mixed model, and agreement between owner and veterinarian-assigned scores using quadratic-weighted Cohen’s kappa and Bland–Altman analysis. Clinician-assigned CIBDAI scores showed a significant overall effect of time (*p* < 0.01), but no pairwise comparison remained significant after correction. Daily monitoring showed a significant nonlinear pattern in the primary diary-only model, although no interval of significant change was identified. In the secondary baseline-inclusive model, the derivative was significantly negative between Days 2 and 5. In within-dog descriptive comparisons, dogs experienced a median of 3.5 days (range: 0–14) with daily CIBDAI scores at or above their individual T0 value. Weighted kappa was 0.680 (95% CI: 0.266–1.000) at T7 and 0.821 (95% CI: 0.690–0.952) at T30. Bland–Altman analysis identified no proportional bias at T7, whereas significant proportional bias was observed at T30, indicating greater divergence between owner and veterinarian-assigned scores at higher CIBDAI values. Daily owner-reported monitoring captured day-to-day fluctuations that would likely have been missed by conventional outpatient assessments performed only at predefined follow-up time points and may therefore support more individualized post-FMT follow-up in dogs with CE.

## Simple Summary

This study evaluated day-to-day clinical changes in 14 dogs with chronic enteropathy during the 30 days following a single fecal microbiota transplantation (FMT). Owners completed a daily clinical diary, while veterinarians assessed the dogs at baseline and on days 7 and 30. Owner- and veterinarian-assigned clinical scores were generally consistent at scheduled follow-up visits, although differences between the two assessments increased at higher Canine Inflammatory Bowel Disease Activity Index (CIBDAI) values at Day 30. Daily owner-reported CIBDAI scores showed a nonlinear pattern over the 30-day follow-up, although no specific interval of significant change was identified. Daily monitoring also identified temporary improvements and periods of clinical worsening that were not always captured during scheduled veterinary visits. These findings show that daily clinical monitoring can provide a more detailed picture of day-to-day changes between scheduled assessments. Daily owner-completed diaries may therefore provide useful complementary information for the clinical follow-up of dogs after FMT.

## 1. Introduction

Chronic enteropathies (CE) are a heterogeneous group of gastrointestinal disorders in dogs, characterized by persistent or recurrent clinical signs and a variable response to treatment [1,2]. Dietary management represents a first-line therapeutic approach in many affected dogs; however, some patients respond only partially or require additional therapeutic interventions [3,4,5,6]. The chronic course of the disease may also negatively affect the quality of life of both affected dogs and their owners [7,8].

Alterations in the intestinal microbiota and in microbial metabolites have been reported in dogs with chronic enteropathy, supporting growing interest in therapeutic strategies aimed at modulating the gut microbiota [9,10,11]. Fecal microbiota transplantation (FMT) has emerged as an innovative therapeutic strategy for the management of CE in dogs [12,13]. Recent veterinary studies have reported clinical improvement following FMT in some dogs with chronic enteropathy, although the available evidence remains heterogeneous and includes case reports, preliminary studies, retrospective case series, and prospective investigations using different treatment protocols [14,15,16,17,18,19,20,21].

Clinical response to FMT has generally been evaluated using standardized clinical disease activity indices recorded at predefined follow-up visits, including Canine Inflammatory Bowel Disease Activity Index (CIBDAI) reassessment 7–21 days after the final FMT with subsequent long-term follow-up [18], a single evaluation approximately 15 days after treatment [22], evaluations at 30, 60, and 90 days [20], or monthly evaluations extending up to 6 months [19]. More recently, Allerton et al. (2026) incorporated repeated owner-reported CIBDAI and fecal score assessments within a 90-day randomized controlled trial, collecting data at Days 2, 7, 28, 60 and 90 [21]. Despite these advances, published studies have relied on intermittent assessments performed at scheduled visits or selected reporting days. Consequently, the day-to-day evolution of clinical signs during the first month following FMT, including transient improvements, short-term fluctuations and early worsening, remains poorly characterized. The primary objective of this study was to evaluate the dynamics of clinical response following a single FMT in dogs with chronic enteropathy over a 30-day observation period using daily owner-reported monitoring. A secondary objective was to evaluate the agreement between daily CIBDAI scores recorded by owners and those assigned by the attending veterinarian during scheduled outpatient follow-up visits (Day 7 and Day 30). We hypothesized that daily home-based monitoring would provide additional longitudinal clinical information, allowing a more detailed characterization of short-term clinical fluctuations and the temporal pattern of response than conventional outpatient assessments alone.

## 2. Materials and Methods

### 2.1. Study Design and Study Population

This prospective, observational, longitudinal pilot study was conducted between April 2024 and June 2025 at the Veterinary Teaching Hospital (VTH) of the University of Parma. Ethical approval was obtained from the Animal Welfare Committee of the University of Parma (Protocol No. 2024-UNPROBA-0000001, 25/01/2024). Written informed consent was obtained from all owners prior to enrollment, and detailed instructions regarding study procedures and home monitoring were provided.

Client-owned dogs of any breed or sex, aged >5 months, diagnosed with non–food-responsive enteropathy (N-FRE) or partial food-responsive enteropathy (pFRE) and undergoing FMT were eligible for inclusion. Dogs were classified as affected by N-FRE or pFRE if they exhibited chronic (≥3 weeks) idiopathic gastrointestinal signs, including chronic diarrhea, recurrent vomiting, nausea, abdominal discomfort or bloating, altered appetite, and/or progressive weight loss, with absent or partial clinical response to at least three or more previous elimination dietary trials, each one lasting at least two weeks.

Prior to enrollment, all dogs underwent a comprehensive diagnostic work-up to exclude secondary gastrointestinal and extra-intestinal causes of clinical signs, including hematology, serum biochemistry, basal serum cortisol concentration or urinary to cortisol-creatinine ratio, canine trypsin-like immunoreactivity, abdominal ultrasonography, and fecal parasitological analysis performed by fecal flotation using Mini-FLOTAC^®^.

To minimize potential confounding factors, owners were instructed not to modify ongoing dietary or pharmacological treatments during the 30-day observation period following FMT. In all cases, FMT was administered as an adjunctive therapy alongside existing medical and dietary management. Previous FMTs were permitted provided they had been performed before study enrolment. Two dogs had undergone a previous FMT within the 60 days preceding inclusion; however, only data related to the first FMT performed during the study period were included in the present analysis.

Dogs were excluded if owners were unable to ensure adequate compliance, defined as failure to complete daily home monitoring and/or failure to attend scheduled follow-up visits. Additional exclusion criteria included worsening of clinical signs requiring any therapeutic modifications during the study period. Significant therapeutic changes, including modifications in diet or administration of antimicrobials, corticosteroids, or other medications potentially affecting gastrointestinal health within 30 days after FMT, resulted in exclusion from the final analysis. Previous administration of systemic antibiotics within the preceding three months and current or previous corticosteroid treatment were not considered exclusion criteria, if treatments remained unchanged throughout the study period. Also, dogs with previously diagnosed exocrine pancreatic insufficiency (EPI) were included provided the dog was receiving appropriate supplementation with pancreatic enzymes and supplementation remained unchanged during the study period.

### 2.2. Fecal Microbiota Transplantation Procedure

All enrolled dogs underwent FMT at the VTH of the University of Parma. A single FMT was administered at inclusion (T0) via retention enema following baseline clinical assessment. Two privately owned healthy adult spayed female dogs (4 and 9 years old), with normal body condition score (BCS) and no history of gastrointestinal disease, served as fecal donors throughout the study. Donors were selected and screened according to the Clinical Guidelines for Fecal Microbiota Transplantation in Companion Animals [12]. In accordance with these guidelines, donors had not received antibiotics or other medical treatments within the preceding 12 months.

Fecal dysbiosis index (DI) values for the two donors were −5.5 for the first donor and −4.9, −3.6, and −4.5 for the second donor during the study period. Only one DI measurement was available for the first donor because the dog was excluded after the initial two fecal transplantations due to owner moving away, whereas the second donor was assessed three times during the study period.

Donor feces were collected within two to five days prior to FMT administration. Samples collected within 24 h before the procedure were stored at 4 °C, whereas samples collected between 24 h and five days prior to administration were frozen at −20 °C until use. To minimize oxygen exposure and preserve microbial viability, fecal samples were stored in sealed containers.

On the day of FMT administration, fecal samples were thawed at room temperature when necessary and diluted with sterile 0.9% saline solution at a 1:2 (*w*/*v*) ratio (feces:saline) under continuous CO_2_ flushing to obtain a homogeneous suspension suitable for rectal enema administration. The suspension was homogenized and filtered through a fine-mesh strainer to remove large particles and subsequently transferred into sterile 60 mL cone-tip syringes.

FMT was administered at a dosage corresponding to approximately 2.5–5 g of feces/kg body weight (BW), following published recommendations for fecal suspension volume according to patient size [20]. Administration was performed using a rubber catheter (18–21 French, selected according to dog size) connected to the syringe and lubricated with a non-bacteriostatic lubricant. The catheter was inserted rectally and advanced cranially, and the fecal suspension was slowly administered as a retention enema. All procedures were performed on an outpatient basis and sedation was not required for any of the dogs enrolled. Following FMT administration, dogs were maintained on cage rest for approximately two hours to prevent premature defecation. Physical activity and food intake were restricted for 4–6 h after the procedure.

If necessary, FMT was repeated after completion of the 30-day observation period based on individual clinical response; however, only data related to the first FMT were included in the present analysis.

### 2.3. Clinical Monitoring and Data Collection

**Figure 1:**
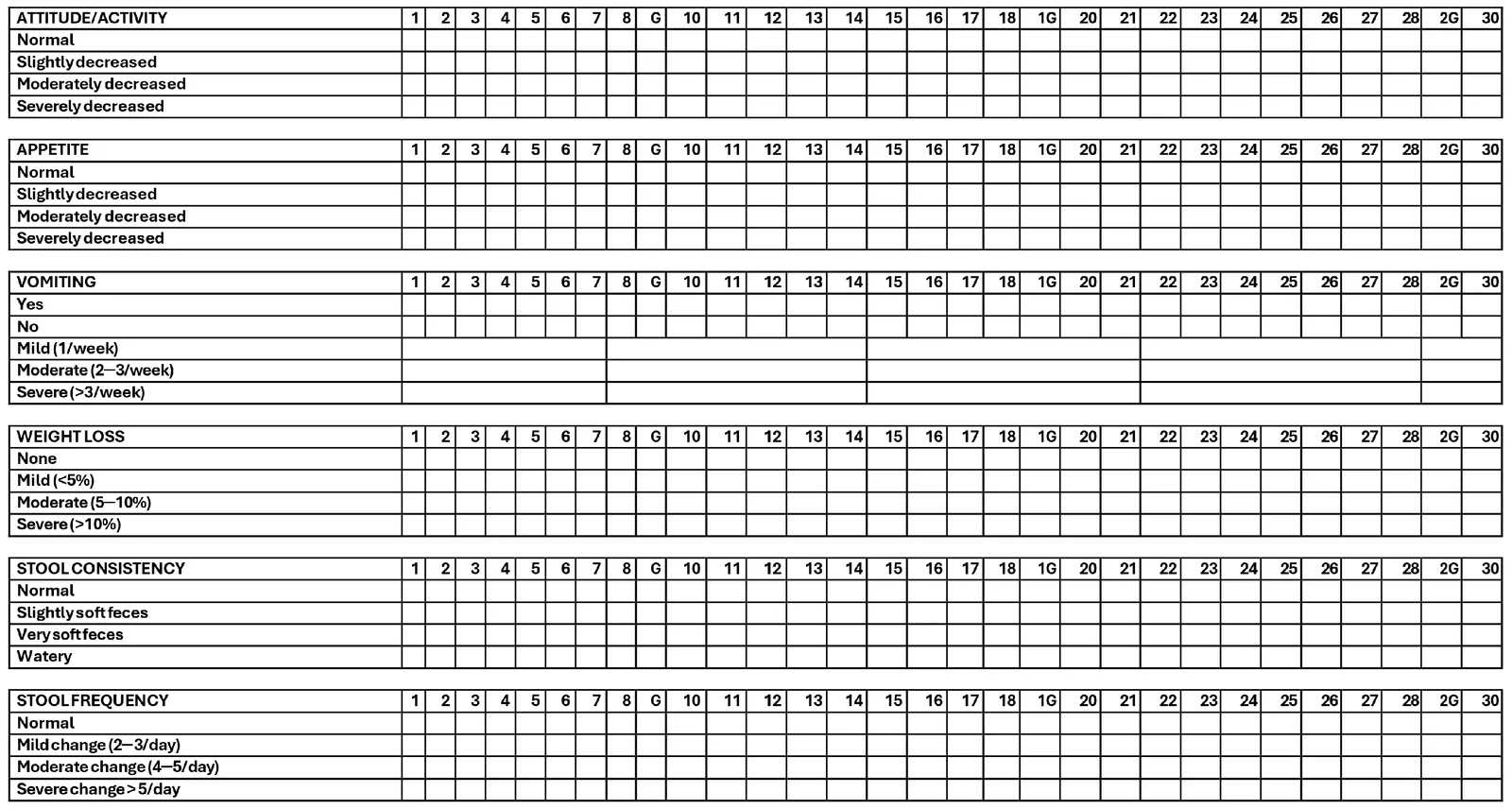
Daily clinical diary completed by owners during the 30-day follow-up after fecal microbiota transplantation (FMT). The diary was adapted from the Canine Inflammatory Bowel Disease Activity Index (CIBDAI) for daily home monitoring and included the standard CIBDAI variables (attitude/activity, appetite, vomiting, weight loss, stool consistency, and stool frequency).

At enrollment (T0), the following data were recorded for all CE dogs: signalment, medical and dietary history, physical examination findings, BW, BCS, CIBDAI, fecal score (FS; Purina Fecal Scoring Chart, Nestlé Purina PetCare), and fecal parasitological analysis. Owners were instructed to complete a daily home-based clinical diary starting the day after FMT administration and continuing for 30 consecutive days (Figure 1).

A paper-based format was selected to facilitate owner compliance. Detailed instructions regarding diary completion were provided at enrollment. The questionnaire was based on the CIBDAI and adapted for daily owner completion. The standard CIBDAI variables were recorded daily to calculate the daily CIBDAI score.

**Figure 2:**
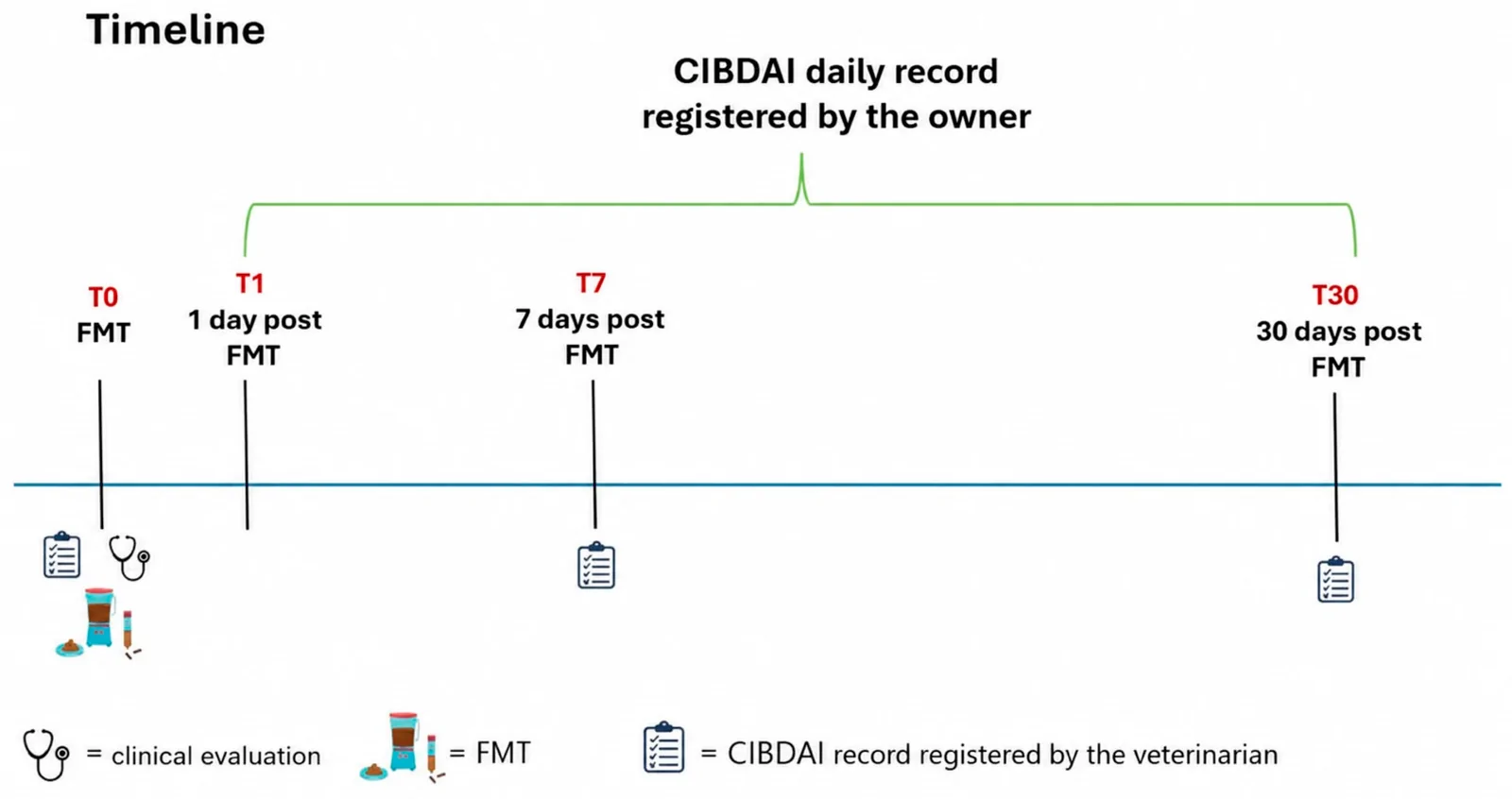
Study timeline. At baseline (T0), dogs underwent clinical evaluation, biological sample collection, and fecal microbiota transplantation (FMT). Beginning the day after FMT (T1), owners completed a daily Canine Inflammatory Bowel Disease Activity Index (CIBDAI) diary for 30 consecutive days. Follow-up evaluations, including clinician-assessed CIBDAI scoring, were performed at T7 and T30.

In addition to daily home monitoring, outpatient clinical re-evaluations were scheduled at Day 7 (T7) and Day 30 (T30) following FMT administration. During these visits, a complete physical examination was performed, including BW measurement and FS assessment. At each visit, the attending clinician independently completed the CIBDAI score (Figure 2). Data obtained during outpatient evaluations at T7 and T30 were compared with daily owner-reported CIBDAI scores for those days once the owner returned the sheet at the end of the follow-up period.

For each dog, daily owner-reported CIBDAI scores were compared with the individual CIBDAI score recorded at T0. Over the 30-day observation period, we calculated the number of days on which the daily CIBDAI was lower than, equal to, or higher than the individual T0 value. Days with scores equal to or higher than T0 were also considered together as days at or above T0. This approach allowed each dog’s clinical course to be evaluated relative to its own initial disease activity. Similarly, the clinician-assigned CIBDAI at T30 was described as lower than, unchanged from, or higher than the individual T0 value.

### 2.4. Statistical Analysis

Statistical analyses were performed using MedCalc (version 23.6.3, Ostend, Belgium) for descriptive and inferential analyses and R (version 4.4.3; R Core Team, 2025) for generalized additive mixed model (GAMM). Baseline demographic and clinical characteristics were summarized descriptively. As this was an observational pilot study without comparison groups, no inferential statistical analyses were performed for baseline variables.

Normality was assessed using the Shapiro–Wilk test. Normally distributed variables are presented as mean ± standard deviation (SD), whereas non-normally distributed variables are reported as median [interquartile range (IQR); range]. Changes in clinician-assessed CIBDAI scores and fecal scores across T0, T7, and T30 were analyzed using the Friedman test followed, when appropriate, by Bonferroni-adjusted Wilcoxon signed-rank tests. Agreement between owner and veterinarian-assigned CIBDAI scores at T7 and T30 was assessed separately at each time point using quadratic-weighted Cohen’s kappa and Bland–Altman analysis. For Bland–Altman analysis, the mean difference between veterinarian and owner-assigned scores was calculated to estimate systematic bias, together with the corresponding 95% confidence interval and 95% limits of agreement. Proportional bias was assessed by linear regression of the paired differences on the mean of the two measurements.

Temporal changes in CIBDAI scores were modelled with a generalized additive mixed model (GAMM) in R (version 4.4.3) using the mgcv package. The diary-only model, based on owner-reported CIBDAI scores from Days 1–30, was considered the primary longitudinal analysis. A secondary baseline-inclusive model additionally incorporated the clinician-assigned CIBDAI measurement at T0 as the pre-treatment reference. Since CIBDAI is a non-negative discrete score, the response was modelled with a negative-binomial distribution (log link); Gaussian and scaled-t alternatives were examined as a sensitivity analysis, and the negative-binomial model was retained based on residual diagnostics and the Akaike Information Criterion (AIC). Follow-up day was modelled with a penalized thin-plate regression spline, s(day), and between-dog variability arising from repeated measurements was accommodated by a dog-specific random intercept. Smoothing parameters were estimated by restricted maximum likelihood (REML), and model adequacy was assessed through residual diagnostics and basis-dimension checks. The owner diaries were complete for all 14 dogs across Days 1–30 and, together with the T0 assigned by the veterinarian, provided 434 observations with no missing data. Evidence for a nonlinear temporal effect was based on the estimated degrees of freedom (edf) of s(day), together with its test statistic and *p*-value and the overall deviance explained. To characterize the direction and timing of change, the first derivative of s(day) was computed with 95% simultaneous confidence intervals; intervals over which the confidence band excluded zero were taken to indicate significant improvement (negative derivative) or worsening (positive derivative), and the day at which the derivative crossed zero identified the point of maximal clinical response. Statistical significance was set at *p* < 0.05 for all analyses.

## 3. Results

### 3.1. Study Population

Twenty dogs diagnosed with CE were initially enrolled between April 2024 and June 2025. Four out of 20 (20%) dogs were excluded because of incomplete owner compliance with the daily clinical diary. The other two dogs were excluded because major therapeutic modifications were required during the 30-day follow-up period. The final study population therefore consisted of 14 dogs.

Baseline clinical characteristics are summarized in Table 1. The study population was heterogeneous with respect to breed, age, body weight, diet, concurrent diseases, and previous treatments. Three dogs had received systemic antibiotics within the preceding three months, whereas three dogs were receiving immunomodulatory treatment at enrollment. Two dogs had previously undergone a single FMT before inclusion, while all remaining dogs received their first FMT as part of the present study. Relevant concurrent diseases included EPI, idiopathic epilepsy, urethral functional outflow obstruction, dermatitis and a previous *Giardia duodenalis* infection (Table 1).

### 3.2. Fecal Microbiota Transplantation Procedure and Immediate Tolerance

All enrolled dogs underwent FMT without procedural complications. No immediate adverse events were observed following transplantation, and fecal retention after enema administration was considered adequate in every patient. Although additional FMTs were performed in some dogs after completion of the study period according to their subsequent clinical course, only data related to the first transplantation were included in the present analysis.

### 3.3. Clinical Response Following FMT

**Figure 3:**
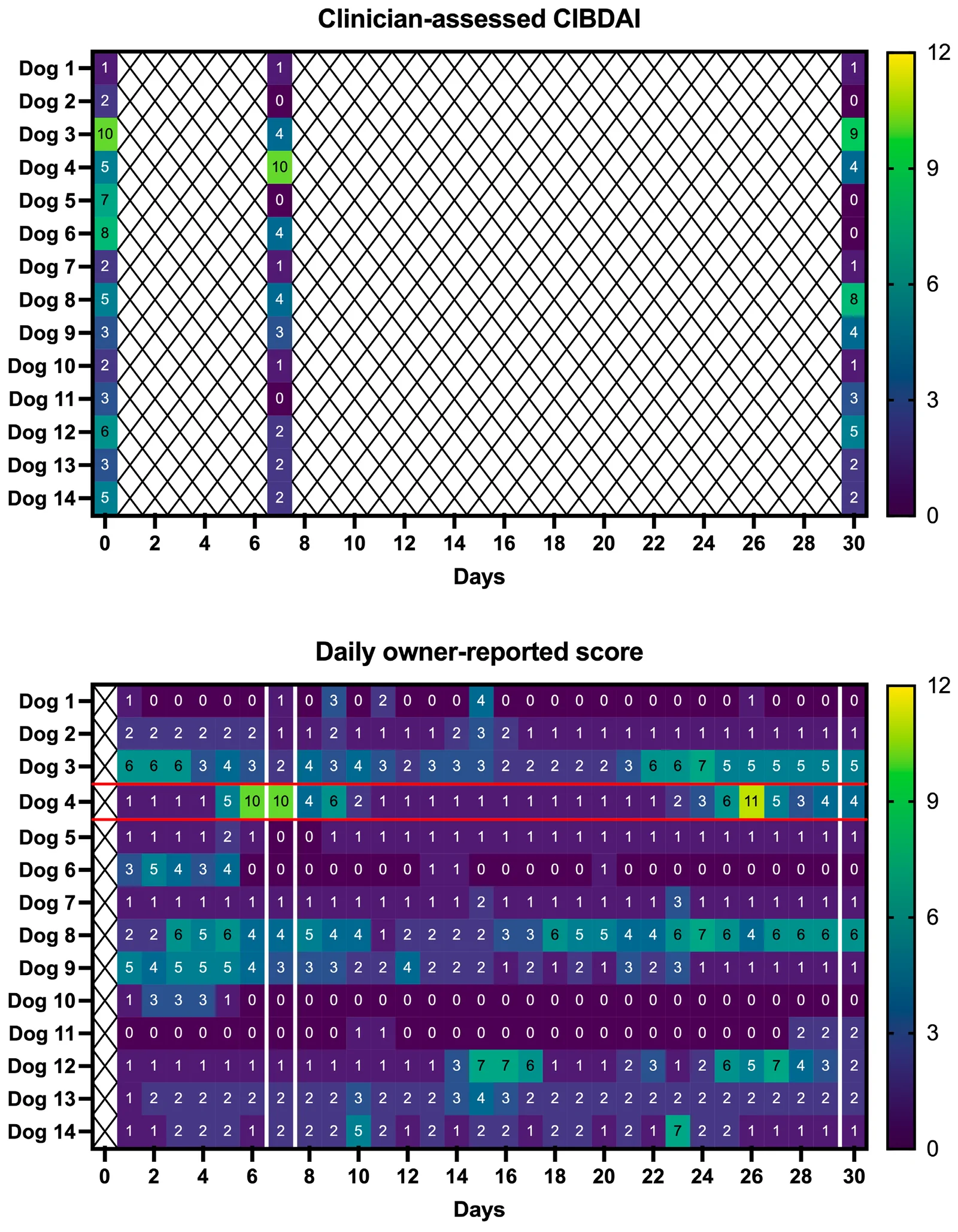
Heatmap representation of Canine Inflammatory Bowel Disease Activity Index (CIBDAI) scores during the 30-day follow-up after fecal microbiota transplantation (FMT) in 14 dogs with chronic enteropathy. The upper panel shows clinician-assigned CIBDAI scores at the scheduled evaluations at baseline (T0), Day 7 (T7), and Day 30 (T30), whereas the lower panel shows daily owner-reported CIBDAI scores recorded from Day 1 to Day 30. Cell colors represent CIBDAI magnitude according to the color scale shown in the figure. Crossed cells indicate time points at which the corresponding assessment was not scheduled and do not represent missing data. In the lower panel T7 and T30 were highlighted by a white outline and correspond to the time points at which both clinician-assigned and owner-reported CIBDAI scores were available; Dog 4 is highlighted by a red outline.

Individual clinical data, including FS and CIBDAI values at T0, T7, and T30, are reported in Table 2. At T0, clinician-assigned CIBDAI scores ranged from 1 to 10, with 7 of 14 dogs presenting a CIBDAI > 3. Among these dogs, CIBDAI scores were lower than their individual T0 values in 6/7 dogs at T7 and in 6/7 dogs at T30. Clinical evaluations performed by the attending clinician at T0, T7 and T30 showed a significant overall effect in CIBDAI scores over time (*p* < 0.01, Kendall’s *W* = 0.42; Figure 3). However, pairwise comparisons did not remain statistically significant after Bonferroni correction. At T7, CIBDAI scores were lower than their individual T0 values in 11 of 14 dogs. At T30, CIBDAI scores were lower than their individual T0 values in 10 of 14 dogs, unchanged in 2 dogs, and higher in 2 dogs (Figure 3). Fecal scores differed significantly across T0, T7, and T30 (Friedman test, *p* = 0.030; Kendall’s *W* = 0.24), with median [IQR] scores of 4.0 [2,3,4,5,6], 2.5 [2,3,4], and 3.0 [2,3,4], respectively. However, none of the pairwise comparisons remained statistically significant after Bonferroni correction (all adjusted *p* > 0.05).

### 3.4. Daily Clinical Monitoring

Daily owner-completed clinical diaries provided a detailed description of the clinical course during the month following FMT. The relationship between clinician-assigned CIBDAI at T30 and the daily clinical course is summarized in Table 3.

Dogs had a median of 3.5 days (range: 0–14) with daily CIBDAI scores at or above their individual T0 value. Four dogs remained below their T0 value throughout the observation period, whereas three dogs had 10 or more days with scores at or above T0.

Six dogs underwent additional FMT after completion of the study period. Among these dogs, five had a clinician-assigned CIBDAI score > 3 at T30, whereas one had a CIBDAI score ≤ 3.

### 3.5. Longitudinal Evolution of Daily Clinical Scores

**Figure 4:**
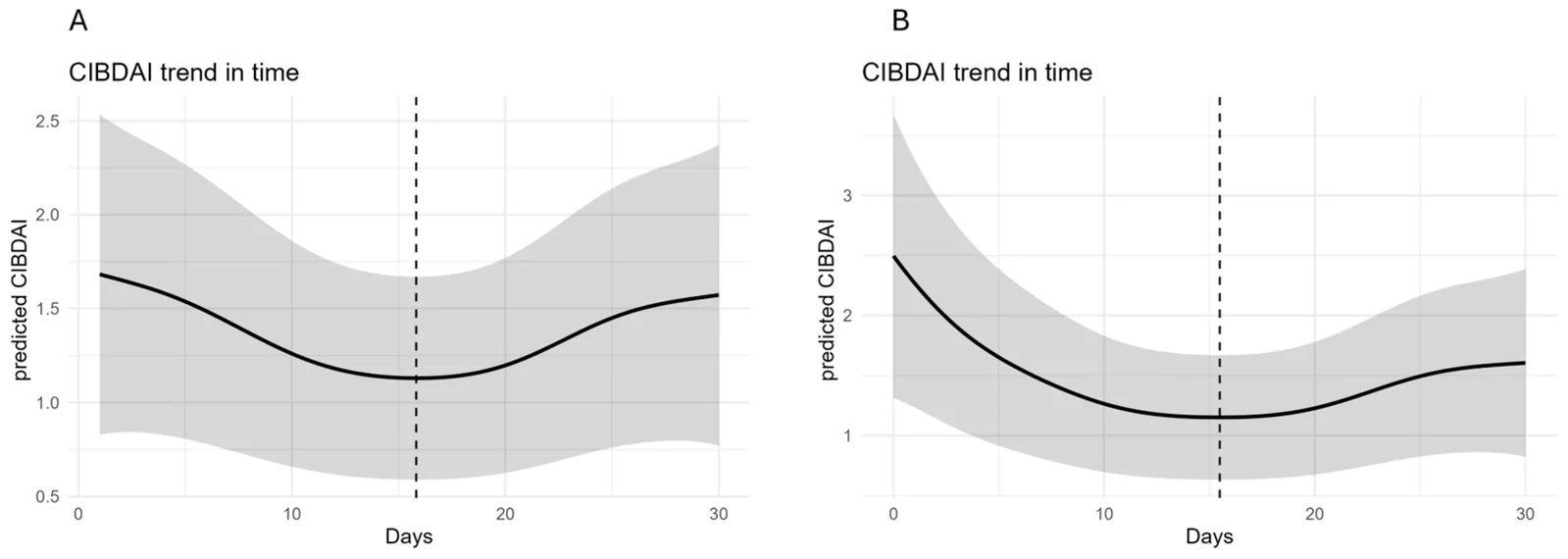
Fitted trajectories of predicted Canine Inflammatory Bowel Disease Activity Index (CIBDAI) scores from the generalized additive mixed models (GAMM). (**A**) Diary-only model based on owner-reported CIBDAI scores from Days 1–30. (**B**) Baseline-inclusive model including the clinician-assigned CIBDAI score at T0 and owner-reported CIBDAI scores from Days 1–30. The grey shaded area represents the 95% confidence interval, the solid line represents the predicted fitted trajectory, and the dashed line indicates Day 15 of treatment.

**Figure 5:**
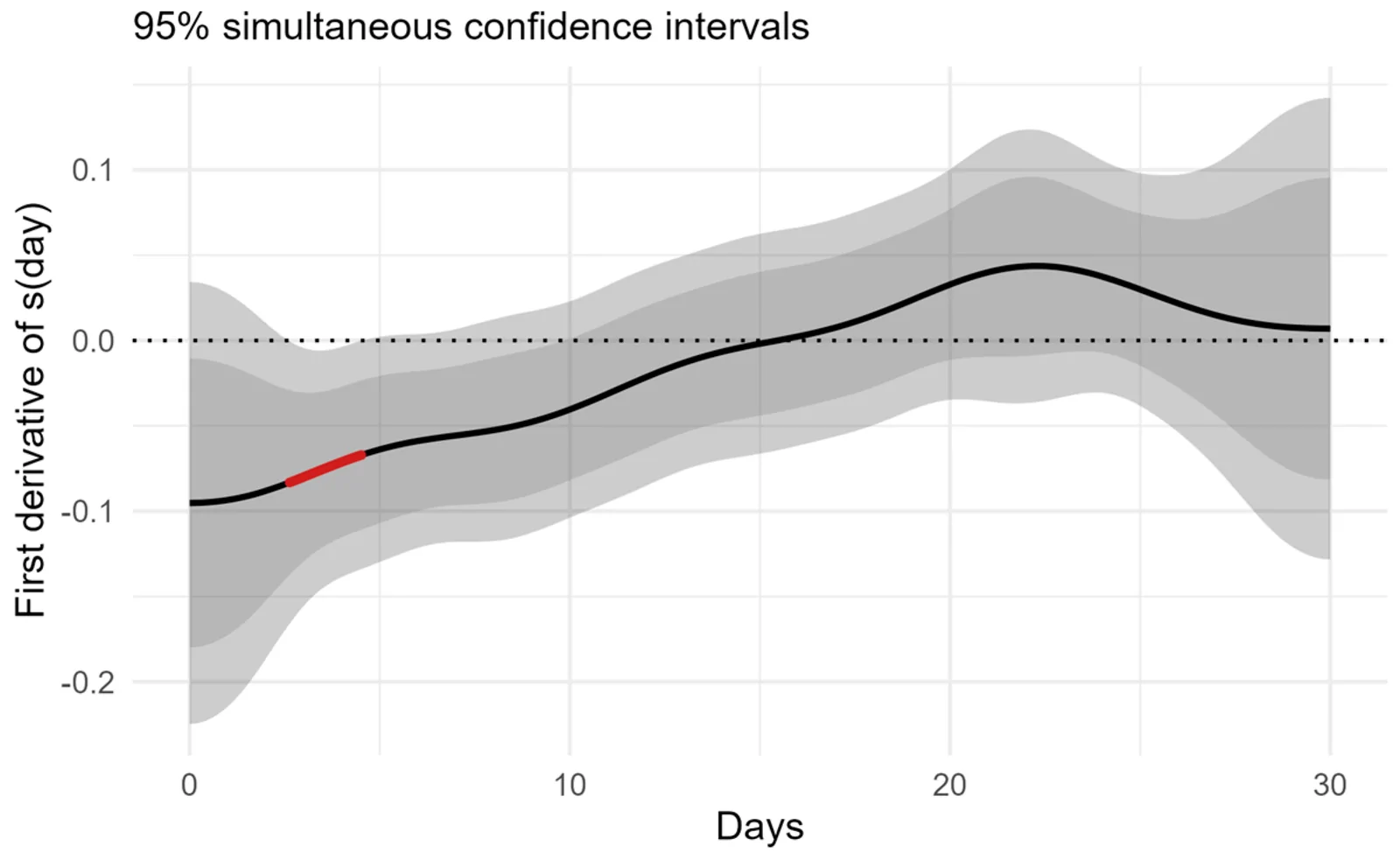
First derivative of the temporal smooth, s(day), from the baseline-inclusive generalized additive mixed models (GAMM) of Canine Inflammatory Bowel Disease Activity Index (CIBDAI) scores, including the clinician-assigned baseline measurement at T0 and owner-reported measurements from Days 1−30. T0 is represented as zero on the temporal axis. The solid line represents the estimated first derivative; the darker and lighter grey bands represent the 95% pointwise and 95% simultaneous confidence intervals, respectively. The horizontal dotted line indicates zero. The red segment identifies the interval during which the first derivative was significantly negative based on the 95% simultaneous confidence interval.

In the primary diary-only analysis (Days 1–30), the nonlinear effect of day was significant (edf = 3.3, Chi-square = 14.4, *p* = 0.007) but the first derivative did not reach significance at any time point (Figure 4A). In the secondary baseline-inclusive analysis incorporating the clinician-assigned T0 measurement, the GAMM confirmed a significant nonlinear effect of follow-up day on CIBDAI scores (s(day): edf = 3.5, Chi-square = 37.6, *p* < 0.001; Figure 4B). The dog-specific random intercept was significant (edf = 12.4, *p* < 0.001), reflecting substantial between-dog differences in overall CIBDAI level, whereas the shape of the temporal trajectory was shared across dogs. The model explained 49.7% of the deviance. In the baseline-inclusive model, the first derivative of s(day) was significantly negative between Days 2 and 5, with the 95% simultaneous confidence interval excluding zero (Figure 5). The point estimate of the derivative remained negative until approximately Day 15, when the fitted trajectory reached its minimum; however, after Day 5 the derivative did not differ significantly from zero. No significant change was identified at later time points.

The average temporal pattern identified by the population-level smooth of the GAMM was consistent with the aggregate day-to-day pattern observed in the daily clinical diaries (Table 3). Since the model included a dog-specific random intercept but a shared temporal shape, differences in individual clinical courses between dogs are described descriptively (Table 3, Figure 3) rather than modelled as dog-specific trajectories. The value of daily home monitoring is illustrated by the clinical course of Dog 4 (Figure 3). Dog 4 had clinician-assigned CIBDAI scores of 5, 10, and 4 at T0, T7, and T30, respectively. Daily owner-reported scores were below the individual T0 value on 23 days and at or above T0 on 7 days. Daily CIBDAI scores remained below baseline for most of the observation period, with transient increases occurring around the T7 assessment and again during the last week of follow-up.

### 3.6. Comparison Between Owner- and Veterinarian-Assessed CIBDAI Scores

**Figure 6:**
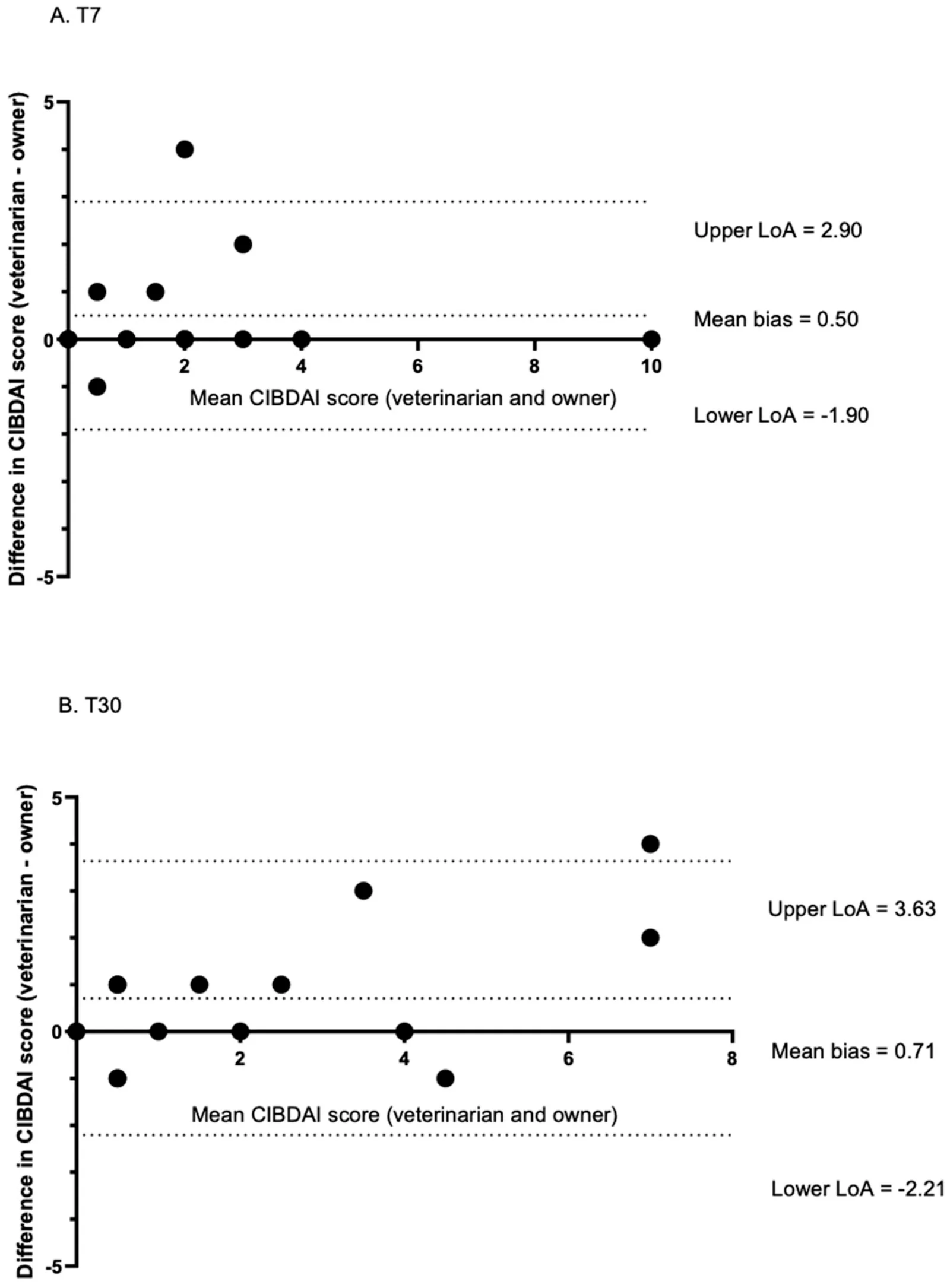
Bland–Altman plots assessing agreement between owner and veterinarian-assigned CIBDAI scores at T7 (**A**) and T30 (**B**). The central dotted line represents the mean difference (bias; veterinarian minus owner), and the upper and lower dotted lines represent the 95% limits of agreement (LoA). Positive differences indicate higher veterinarian-assigned than owner-assigned CIBDAI scores. Significant proportional bias was detected at T30 (*p* = 0.002), but not at T7 (*p* = 0.410). Overlapping points represent identical paired observations.

Median CIBDAI scores assigned by the attending veterinarian and owners were 2.0 (IQR: 1–4; range: 0–10) and 1.0 (IQR: 0–2; range: 0–10) at T7, and 2.0 (IQR: 1–4; range: 0–9) and 1.5 (IQR: 1–4; range: 0–6) at T30, respectively. Agreement between owner and veterinarian-assigned CIBDAI scores was assessed using quadratic-weighted Cohen’s kappa. Weighted kappa was 0.680 (95% CI: 0.266–1.000) at T7 and 0.821 (95% CI: 0.690–0.952) at T30. Bland–Altman analysis showed mean veterinarian–owner differences of 0.50 points (95% CI: −0.21 to 1.21; limits of agreement: −1.90 to 2.90) at T7 and 0.71 points (95% CI: −0.15 to 1.57; limits of agreement: −2.21 to 3.63) at T30. No significant proportional bias was detected at T7 (*p* = 0.41), whereas significant proportional bias was observed at T30 (slope = 0.385, 95% CI: 0.167–0.603; *p* < 0.01) (Figure 6).

## 4. Discussion

The present study suggests that daily owner-reported clinical monitoring provides additional information on the temporal evolution of clinical signs that may not be captured by conventional outpatient evaluations performed only at predefined follow-up time points. In this cohort, FMT was well tolerated, with no immediate adverse events observed, and CIBDAI scores generally decreased during follow-up; however, the absence of a control group prevents these clinical changes from being attributed specifically to FMT. The main novelty of the present study lies in the use of a structured daily owner-completed clinical diary. The primary diary-only analysis identified a significant nonlinear temporal pattern, although no specific interval of significant change was detected. In the secondary baseline-inclusive model, a significant early decrease in CIBDAI scores was identified between Days 2 and 5. At the scheduled clinical assessments, clinician-assigned CIBDAI scores at T30 were lower than the individual T0 values in 10 of 14 dogs. These findings are consistent with previous veterinary reports describing improvement of CIBDAI following FMT administration [14,18,20,23]. Clinical improvement following FMT has also been reported in dogs with chronic idiopathic large-bowel diarrhea, although the study populations and treatment protocols differ from those used in dogs with chronic enteropathy [24]. Fecal scores showed an overall variation across the scheduled assessments; however, no pairwise comparison remained statistically significant after Bonferroni correction, precluding identification of a specific time point associated with a significant change in fecal consistency. Furthermore, daily monitoring showed that the clinical course during the 30-day follow-up did not follow a linear temporal pattern. The primary diary-only model confirmed a nonlinear temporal pattern, although no specific interval of significant change was identified. The secondary baseline-inclusive model identified a significant early decrease between Days 2 and 5; however, this finding should be interpreted cautiously because the T0 measurement was clinician-assigned, whereas the subsequent daily measurements were owner-reported. The day-to-day worsening captured by the diaries is therefore described qualitatively. Such temporal fluctuations would not have been adequately captured by scheduled outpatient evaluations alone. The marked inter-individual variability observed in the present cohort should also be considered in the context of the heterogeneity of clinical indications and FMT protocols currently reported in veterinary practice [25].

Some dogs whose clinician-assigned CIBDAI at T30 remained unchanged or was higher than the individual T0 value still experienced prolonged periods with lower daily CIBDAI scores. Dog 11 provides a particularly illustrative example: the clinician-assigned CIBDAI was 3 at T0, decreased to 0 at T7, and returned to 3 at T30, whereas daily owner-reported CIBDAI scores remained below the individual T0 value throughout the 30-day observation period. Similarly, Dogs 8 and 9 had 16 and 18 days, respectively, with daily CIBDAI scores below their individual T0 values despite having higher clinician-assigned scores at T30. Conversely, some dogs with a lower clinician-assigned CIBDAI at T30 still had occasional days during follow-up when their owner-reported score returned to or exceeded the individual T0 value.

Dog 4 provides a representative example of this phenomenon. The increase at T7 was captured by both the owner and the veterinarian, who each assigned a CIBDAI score of 10; however, the daily diary additionally documented fluctuations during the final week of follow-up that were not represented by the scheduled T30 assessment alone. Daily owner-reported CIBDAI scores were below the individual T0 value on 23 days and at or above T0 on 7 days. It should also be noted that inclusion in the present study was not based solely on CIBDAI scores. Dogs were considered candidates for FMT according to the overall clinical assessment, including medical history and persistent gastrointestinal signs judged by the attending clinician to warrant microbiota-directed therapy, even when CIBDAI scores were low.

Quadratic-weighted Cohen’s kappa indicated agreement between owner and veterinarian-assigned CIBDAI scores at both T7 and T30, although the estimate at T7 was characterized by a wide confidence interval. Bland–Altman analysis showed small positive mean veterinarian-owner differences at both time points, with 95% confidence intervals including zero. However, significant proportional bias was identified at T30, indicating that the difference between veterinarian and owner-assigned scores increased as mean CIBDAI values increased. This suggests that the two assessments may diverge at higher disease activity, with owners tending to assign lower scores than veterinarians at higher CIBDAI values. Owner and veterinarian-assigned scores should therefore not be considered fully interchangeable. Nevertheless, daily owner-completed diaries may provide complementary information on clinical evolution between scheduled veterinary visits. Consequently, daily owner-based symptom monitoring may represent a practical tool for long-term clinical follow-up. These findings agree with the observations reported by Lyngby et al. (2024) [8], according to whom owner involvement represents a crucial component in the management of chronic diseases. Structured clinical diaries appear to enrich follow-up evaluations by enabling earlier recognition of critical clinical phases that may otherwise escape conventional clinical assessment. Such an approach becomes possible only through active owner participation in the daily monitoring of dogs affected by chronic enteropathy [7]. Owner compliance with daily diary completion was high, with 80% of owners who were offered participation completing the daily questionnaire throughout the 30-day study period.

Consistent with previous veterinary studies, no immediate procedure-related adverse events were observed following FMT administration in the present cohort [12,13,14,26]. Broader canine studies in acute gastrointestinal disease have likewise reported that FMT can be administered with acceptable short-term tolerability, although its clinical effects have not been uniform across study populations [27,28,29,30]. The need for repeated FMT administration in a subset of dogs is in agreement with previous veterinary experience suggesting that multiple FMT procedures may be required in selected patients [12,18]. However, the optimal timing and frequency of repeated FMT remain uncertain, and microbiota-directed interventions are still best considered within the broader, often multimodal management of canine chronic enteropathy [31,32].

### Study Limitations

The present study has several limitations. First, the small sample size limits the statistical power of the study and the generalizability of the findings. Second, the absence of a placebo-treated control group prevents definitive attribution of the observed clinical improvements exclusively to FMT. Moreover, because daily CIBDAI scores were not evaluated in healthy dogs, dogs with clinically stable chronic enteropathy, or dogs receiving alternative treatments, it remains unclear whether the observed temporal fluctuations reflect the natural day-to-day variability of the disease or are specifically associated with FMT. In the baseline-inclusive GAMM, the T0 value was assigned by the clinician, whereas all subsequent daily scores were reported by owners. Because the agreement analysis indicated that owner and veterinarian-assigned CIBDAI scores were not fully interchangeable, this differently sourced baseline observation may have contributed to the estimated early decline. Accordingly, the diary-only model, based exclusively on owner-reported scores, was considered the primary analysis, whereas the baseline-inclusive model was retained as a secondary analysis providing a pre-treatment reference. The number of days with owner-reported CIBDAI scores below or at/above the individual T0 value should be interpreted as a within-dog descriptive measure and is not directly comparable across dogs, because the opportunity to cross the individual baseline depends on the baseline score and the bounds of the CIBDAI scale; moreover, crossing this threshold does not necessarily represent a clinically meaningful change.

In addition, treatment protocols, dietary management, and clinical assessments were not fully standardized among dogs. Two dogs had undergone a previous FMT within the 60 days preceding enrollment. A residual effect of these previous procedures on baseline clinical status or on the subsequent clinical course cannot be excluded and represents a potential confounding factor. Moreover, two dogs were excluded after enrollment because worsening clinical signs required therapeutic modifications during the 30-day follow-up period. Because this exclusion was based on post-enrollment clinical outcome, dogs with less favorable clinical trajectories may have been preferentially excluded, potentially leading to an underestimation of the frequency or magnitude of clinical worsening during follow-up. Another limitation is that fecal microbiota composition was not evaluated before and after FMT in recipient dogs, preventing assessment of whether the observed clinical changes were associated with corresponding changes in the intestinal microbiota. This is particularly relevant because clinical improvement following FMT does not necessarily coincide with consistent changes in fecal microbial community composition, as reported in some canine studies [23,29]. Despite these limitations, the study highlights the added value of structured daily owner-completed clinical diaries for characterizing the temporal evolution of the clinical course during follow-up after FMT.

## 5. Conclusions

In conclusion, the results of the present study suggest that the clinical course of dogs with chronic enteropathy following a single FMT does not follow a linear temporal pattern. In the absence of a control group, these temporal changes cannot be attributed specifically to FMT and may also reflect the natural day-to-day variability of the disease. This temporal pattern was identified through daily owner-reported CIBDAI monitoring performed in the home environment. Such a diary-based approach provides an additional perspective for evaluating clinical evolution during follow-up, allowing detection of day-to-day fluctuations that may not be captured by outpatient evaluations performed only at predefined time points.

Daily owner-reported monitoring provided additional information on short-term clinical fluctuations that was not fully captured by scheduled outpatient assessment. The monitoring strategy adopted in this study may therefore support more individualized clinical follow-up of dogs undergoing FMT by providing clinicians with a more detailed picture of short-term changes between scheduled assessments. Furthermore, this approach may promote greater owner involvement in the therapeutic management of dogs affected by chronic enteropathy.

## Figures and Tables

**Table 1:** Baseline clinical and treatment characteristics of dogs with chronic enteropathy (CE) undergoing fecal microbiota transplantation (FMT). Data include breed, serum albumin concentration, diet at enrollment, antibiotic exposure within the preceding 3 months, immunomodulatory treatment at enrollment, relevant comorbidities, and the number of FMT procedures performed before baseline (T0).

Dog	Breed	Albumin (g/dL)	Diet	Antibiotics (≤3 Months)	Immunomodulatory	Comorbidities	*n* FMT pre T0
Dog 1	Maltese	3.24	Home-cooked diet	No	No	Dermatitis	0
Dog 2	Chihuahua	2.60	Home-cooked diet	No	Prednisolone/ Cyclosporine	Idiopathic epilepsy	0
Dog 3	Basset Hound	3.05	Home-cooked diet	No	No	Previous giardiasis	0
Dog 4	Labrador Retriever	2.63	Purina HA Hypoallergenic	Yes	No	Urethral FOO	1
Dog 5	French Bulldog	2.78	Purina HA Hypoallergenic	No	No	None	0
Dog 6	Bracco Italiano	2.42	Exclusion Pork & Rice	No	No	None	1
Dog 7	Australian Shepherd	3.31	Purina HA Hypoallergenic	No	No	None	0
Dog 8	Mixed breed	2.85	Purina HA Hypoallergenic	Yes	Prednisolone	None	0
Dog 9	Bichon Havanese	3.26	Exclusion Pork & Rice	No	No	None	0
Dog 10	Golden Retriever	2.12	Exclusion Pork & Rice	No	No	EPI	0
Dog 11	Mixed breed	3.75	Home-cooked diet	No	No	None	0
Dog 12	Dobermann Pincher	2.22	Farmina Intestinal UltraCare Low Fat	No	No	None	0
Dog 13	Samoyed	2.25	Exclusion Pork & Rice	No	No	None	0
Dog 14	German Shepherd	3.2	Hill’s Intestinal	Yes	Prednisolone	EPI, Dermatitis	0

Abbreviations: FOO = functional outflow obstruction; EPI = exocrine pancreatic insufficiency.

**Table 2:** Individual fecal score (FS) and Canine Inflammatory Bowel Disease Activity Index (CIBDAI) values at baseline (T0), Day 7 (T7), and Day 30 (T30) following fecal microbiota transplantation (FMT), together with information on additional FMT procedures performed after T30.

Dog	T0		T7		T30		FMT Post T30
	FS	CIBDAI	FS	CIBDAI	FS	CIBDAI	
Dog 1	2	1	2	1	2	1	No
Dog 2	3	2	2	0	4	0	No
Dog 3	7	10	5	4	6	9	Yes
Dog 4	2	5	2	10	2	4	Yes
Dog 5	7	7	1	0	1	0	No
Dog 6	7	8	5	4	2	0	No
Dog 7	4	2	3	1	2	1	No
Dog 8	4	5	3	4	6	8	Yes
Dog 9	4	3	6	3	3	4	Yes
Dog 10	2	2	2	1	2	1	No
Dog 11	2	3	2	0	4	3	No
Dog 12	6	6	4	2	5	5	Yes
Dog 13	4	3	2	2	3	2	Yes
Dog 14	6	5	4	2	3	2	No

**Table 3:** Relationship between the clinical course assessed at T30 and the daily clinical course during the 30-day follow-up after a single fecal microbiota transplantation (FMT). CIBDAI T0 represents the baseline disease activity score assigned by the attending clinician before FMT. The clinician-assigned CIBDAI at T30 was compared with the individual T0 value and described as lower, unchanged, or higher. Daily owner-reported scores were summarized as the number of days below, equal to, or above the individual T0 value. Days at or above T0 represent the combined number of days on which the daily CIBDAI was equal to or higher than the individual T0 score.

Dog	CIBDAI at T0	CIBDAI at T30 vs. T0	Days Below T0	Days Equal to T0	Days Above T0	Days at or Above T0
Dog 1	1	Unchanged	24	3	3	6
Dog 2	2	Lower	20	9	1	10
Dog 3	10	Lower	30	0	0	0
Dog 4	5	Lower	23	2	5	7
Dog 5	7	Lower	30	0	0	0
Dog 6	8	Lower	30	0	0	0
Dog 7	2	Lower	28	1	1	2
Dog 8	5	Higher	16	4	10	14
Dog 9	3	Higher	18	5	7	12
Dog 10	2	Lower	27	0	3	3
Dog 11	3	Unchanged	30	0	0	0
Dog 12	6	Lower	25	2	3	5
Dog 13	3	Lower	26	3	1	4
Dog 14	5	Lower	28	1	1	2

## Data Availability

The data presented in this study are available from the corresponding author upon reasonable request. The data are not publicly available due to privacy considerations related to client-owned animals.

## Abbreviations

The following abbreviations are used in this manuscript:

BCS	Body Condition Score
BW	Body Weight
CE	Chronic Enteropathy
CIBDAI	Canine Inflammatory Bowel Disease Activity Index
DI	Dysbiosis Index
EPI	Exocrine Pancreatic Insufficiency
FMT	Fecal Microbiota Transplantation
FS	Fecal Score
GAMM	Generalized Additive Mixed Model
IQR	Interquartile Range
N-FRE	Non-Food-Responsive Enteropathy
pFRE	Partial Food-Responsive Enteropathy
SD	Standard Deviation
VTH	Veterinary Teaching Hospital
